# La place de la chirurgie à ciel ouvert dans la reprise de la sténose urétérale étendue, ancienne et post infectieuse: à propos d’un cas

**DOI:** 10.11604/pamj.2021.38.276.26984

**Published:** 2021-03-17

**Authors:** Tresor Kibangula Kasanga, Eric Mbuya Musapoudi, Augustin Kibonge Mukakala, Serges Ngoy Yumba, François Katshitsthi Mwamba, Patrick Ciza Zihairwa, Nathalie Dinganga Kapessa, Mbey Mukaz

**Affiliations:** 1 Département de Chirurgie, Unité d´Urologie, Faculté de Médecine, Clinique Universitaire de Lubumbashi, Université de Lubumbashi, Lubumbashi, Province du Haut-Katanga, République Démocratique du Congo,; 2 Département de Chirurgie, Faculté de Médecine, Clinique Universitaire de Bukavu, Université officielle de Bukavu, Bukavu, République Démocratique du Congo,; 3 Département de Chirurgie, Faculté de Médecine, Clinique Universitaire de Kolwezi, Kolwezi, République Démocratique du Congo

**Keywords:** Chirurgie à ciel ouvert, sténose urétérale étendue, infection, à propos d’un cas, Open surgery, extended ureteral stricture, infection, case report

## Abstract

Notre travail vise à rapporter, la place de la chirurgie à ciel ouvert dans la reprise d´une sténose urétérale étendue, ancienne et post infectieuse d´une part, et d´autre part, d´apporter notre expérience thérapeutique, cela à travers l´observation d´un patient âgé de 38 ans qui nous avait consulté dans notre service d´urologie des cliniques universitaires de Lubumbashi, pour douleur lombaire gauche, brûlures mictionnelles, fièvre sans horaire, Le bilan clinique et paraclinique, mettaient en évidence la sténose urétérale étendue. Une dérivation urinaire temporaire (urétérostomie gauche) était réalisée. Il a été repris, pour une urétérectomie suivie d´une réimplantation urétéro-vésicale gauche. Les suites postopératoires ont été simples, le patient a été revu 3 mois après l´intervention, il n´avait aucune plainte, la créatinine contrôle était normale, l´échographie et l´uroscan étaient dans les normes.

## Abstract (en)

The purpose of this study is to highlight the role of open surgery in the treatment of patients with a history of extended and postinfectious ureteral stricture as well as our experience with therapy. The study involved a 38-year old patient presenting at the urology service of the University Clinics of Lubumbashi with left lumbar pain, burning sensation during urination and fever. Clinical and paraclinical assessment showed extended ureteral stricture. Temporary urinary diversion (left ureterostomy) was performed followed by ureterectomy and left ureterovesical reimplantation. The postoperative course was simple. Three months after surgery the patient had no complaints, creatinine levels were normal, ultrasound and uroscan results were normal.

## Introduction

Le traitement le plus efficace de sténose de l´uretère distal ancienne et étendue est la correction par la chirurgie ouverte avec un taux de succès supérieur à 80% ou 100% [1]. Cependant, l´agressivité de la chirurgie ouverte incite à proposer en première intention un traitement endoscopique, dont les avantages sont une faible morbidité et le respect de la vascularisation urétérale et dont l´inconvénient est de laisser en place les lésions péri-urétérales [2]. Et en cas d´échec de cette dernière on recourt toujours à la précédente [3]. La sténose urétérale est une diminution pathologique permanente et définitive du calibre de la lumière urétérale [4]. Les sténoses de l´uretère posent des problèmes très différents suivant leurs étiologies, sur le plan diagnostique et surtout thérapeutique [5]. Les étiologies des obstructions de l´uretère sont extrêmement nombreuses, sont intimement liées aux pathologies, aux traitements chirurgicaux de l´uretère, aux maladies des organes de voisinage intra et extra péritonéaux [5]. Cet article présente l´intérêt de la chirurgie à ciel ouvert dans la reprise de la sténose urétérale ancienne et étendue.

## Patient et observation

Il s´agissait d´un patient âgé de 38 ans qui nous avait consulté dans notre service d´urologie des cliniques universitaires de Lubumbashi pour douleur lombaire gauche de forte intensité irradiant vers le testicule homolatéral insomniante, sans aucun facteur déclenchant ou d´accalmie depuis 4 mois. Dans ses antécédents, il existait une notion de brûlures mictionnelles depuis 6 mois, il avait présenté une crise similaire antérieure il y a 2 ans. L´examen physique avait mis en évidence une sensibilité des points urétéraux supérieurs et moyens gauche, à l´ébranlement rénal, celui-ci avait réveillé la douleur dans la région lombaire gauche, avec un contact lombaire mis en évidence, autres systèmes.

**Figure 1:**
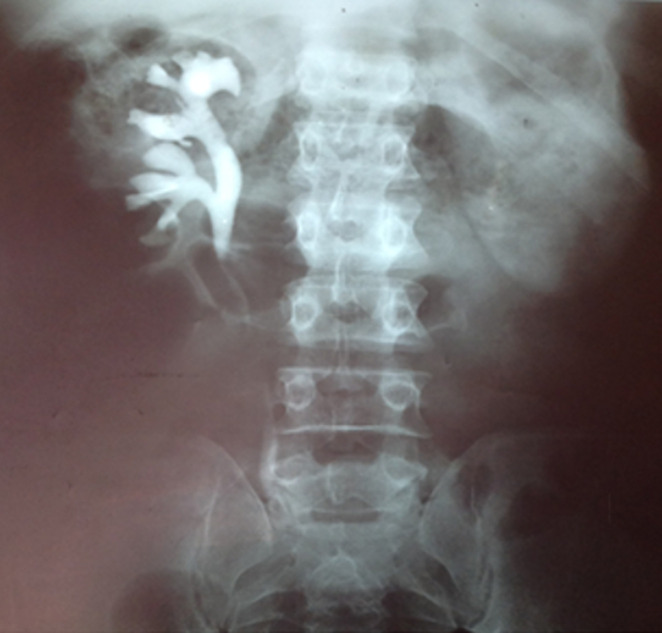
image de l´urographie intraveineuse (UIV) montrant une urétérohydronéphrose droite associée

**Figure 2:**
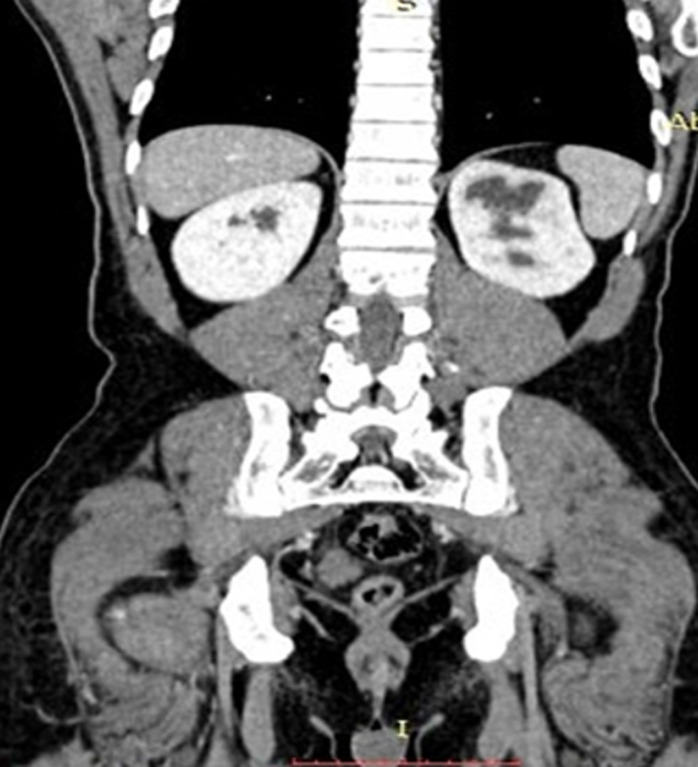
image scannée abdominale et uroscan montrant l´urétérohydronéphrose droite

Sur le plan biologique : la créatinine 1,47 mg%, urée 27 mg, Mg+ 2,2 mg, Ca + 10,3 mg, Gs O Rh+, Hb 16,7g%, Hte 48%, GR 5.440.000/mm^3^, GB 4600/mm^3^, VS 4mm/h, FL : GN 75%, B 24%, VGM 88um^3^, TGMHb 30,6pg, IDR 9mm et l´ECBU avait isolé le *Pseudomonas sp* sensible aux Imipenèmes. L´échographie de l´arbre urinaire avait mis en évidence une urétérohydronéphrose gauche, pas de calcul objectivé. L´urographie intraveineuse (UIV) avait mis en évidence une urétérohydronéphrose gauche associée (Figure 1). Les Scanner abdominal et uroscan avaient montré l´urétérohydronéphrose gauche associée à une hypotonie pyelocalicielle gauche, comme diagnostic qui avait été retenu (Figure 2). L´indication d´une exploration chirurgicale par la laparotomie médiane sous ombilicale, il existait une dilatation en amont de la sténose de l´uretère juxta-vésical gauche. Une dérivation urinaire temporaire a été réalisée (urétérostomie gauche) par la sonde urétérale simple. En période postopératoire, il a été pris en charge par une équipe mixte associant les réanimateurs et les chirurgiens. Il avait bénéficié d´une antibioprophylaxie, un apport liquidien en perfusion, une analgésie par voie parentérale.

**Figure 3:**
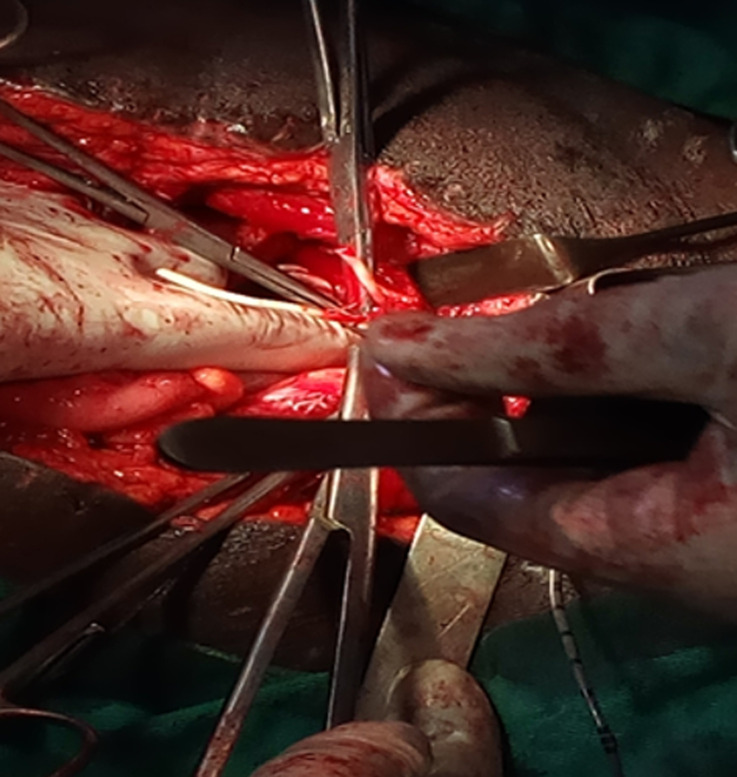
confection de la réimplantation urétéro-vésicale

Son évolution était marquée par l´hématurie macroscopique totale, une pyurie, le hoquet, les vomissements, les constipations, motivent sa reprise au 20^e^ jour postopératoire. La portion sténosée mesurait environ 4cm d´étendue, situait au niveau de la région juxta vésicale gauche, celle-ci a été sectionnée (urétérectomie segmentaire) suivi d´une réimplantation uretèro-vésicale gauche selon la technique de Paquin (Figure 3) sur une sonde urétérale. Les suites postopératoires ont été simples, l´examen anatomo-pathologique de la pièce de l´urétérectomie avait mis en évidence une inflammation chronique non spécifique, le patient a été revu 3 mois après l´intervention, il n´avait aucune plainte, la créatinine contrôle était normale, l´échographie et l´uroscan étaient dans les normes.

## Discussion

Le traitement traditionnel d´une sténose urétérale se fait par la chirurgie à ciel ouverte avec anastomose urétéro-urétérale, urétéro-vésicale ou urétéro-iléale, l´urétérale, et la dérivation urinaire, elles-mêmes source de sténose [6]. Ceci rejoint notre approche thérapeutique de recourir à la chirurgie à ciel ouverte étant donné que dans notre contrée la pratique d´endo-urologie est très peu développée et cette dernière est vouée à l´échec devant une sténose étendue, ancienne et une reprise chirurgicale post urétérostomie. Le diagnostic est fait souvent par l´imagerie qui montre l´arrêt de progression du produit de contraste dans l´uretère sur le cliché d´UIV à l´uro-TDM, à l´UPR ou à la pyélographie descendante [7]. Certes le diagnostic imagerique seul ne suffit pas, pour une prise en charge holistique du patient. Il faut toujours associer la biologie et l´anatomopathologie pour la certitude du diagnostic, comme dans notre cas de figure. La prédominance masculine est nette dans toutes les séries [8]. Comme c´était le cas pour notre patient qui était du sexe masculin. Cette prédominance de sexe ne sera pas significative parce qu´il s´agit d´une observation. La douleur lombaire était le motif de consultation le plus fréquent soit 40 % des cas Galifer *et al*. qui ont trouvé une symptomatologie douloureuse dans 35,6% des cas [8]. Comme c´était le cas pour notre patient.

En règle générale, un traitement endo-urologique (par pose d´une sonde double J) était réalisé en première intention en présence d´une sténose distale en particulier siégeant au niveau de l´implantation urétéro-vésicale, précoce (< 3 mois) et courte (< 2 cm). Dans les autres situations ou en cas d´échec des techniques endoscopiques ou percutanées, une correction chirurgicale à ciel ouvert était réalisée [9], ceci rejoint notre approche thérapeutique, la sténose urétérale, était supérieure à 2cm, ancienne (plus de 3 mois) et c´était une reprise ou un cas d´échec. L´analyse objective des résultats de la chirurgie à ciel ouvert à la chirurgie endoscopique ou percutanée montre la supériorité des techniques classiques [10]. La réimplantation urétérale selon la technique de Paquin paraît tout à fait adaptée à la reconstruction de l´appareil urinaire [11] ; ce qui rejoint notre technique utilisée. Par rapport à la technique de réimplantation vésicale, que ça soit extra ou trans-vésicale, il n´y a aucune publication sur une évaluation à long terme dans deux groupes similaires, il n´y a pas de différence significative dans l´incidence globale des complications urologiques majeures [11]. Les progrès de l´endo-urologie, le développement des ballonnets de dilatation à haute pression, la pose de la sonde JJ de longue durée et la pose d´une endoprothèse expansive dans l´uretère ont fait une alternative à la chirurgie classique [6]. Mais ne la remplace pas totalement, surtout dans nos milieux ou l´endo-urologie est moins développée et très souvent ce sont des sténoses étendues, anciennes voir mêmes les cas des récidives voilà pourquoi on recourt à la chirurgie classique première.

## Conclusion

La place de choix de la chirurgie à ciel ouvert dans la reprise d´une sténose urétérale étendue, ancienne post infectieuse et même en cas d'échec des techniques endoscopiques ou percutanées, est acceptée dans les pays nantis avec beaucoup de controverses et mieux adaptée dans les pays en voie de développement ou pauvres surtout dans notre contrée.
